# Managing and treating Sydenham chorea: A systematic review

**DOI:** 10.1002/brb3.3035

**Published:** 2023-05-07

**Authors:** Samiuddin Tariq, Faizan Niaz, Summaiyya Waseem, Taha Gul Shaikh, Syed Hassan Ahmed, Muhammad Irfan, Abdulqadir J. Nashwan, Irfan Ullah

**Affiliations:** ^1^ Dow Medical College Dow University of Health Sciences Karachi Pakistan; ^2^ Department of Internal Medicine Wellstar Health System Spalding Hospital Griffin Georgia USA; ^3^ Hamad Medical Corporation Doha Qatar; ^4^ Kabir Medical College Gandhara University Peshawar Khyber Pakhtunkhkwa Pakistan

**Keywords:** Sydenham's chorea, group a beta‐hemolytic *Streptococcus*, gamma‐aminobutyric acid, acute rheumatic fever

## Abstract

**Introduction:**

Sydenham's chorea (SC), prevalent in developing countries and occasionally affecting developed ones, poses a clinical challenge due to the lack of systematic guidelines for diagnosis and treatment. Resulting from Group A Beta‐Hemolytic *Streptococcus* infection, SC presents various symptoms. This review aims to collect and evaluate available data on SC management to propose a cohesive treatment plan.

**Methods:**

We searched PubMed, the Cochrane Library, Google Scholar, and ClinicalTrials.gov for literature on SC management from inception until 24th July 2022. Studies were screened by titles and abstracts. Cochrane Collaboration's Risk of Bias tool (RoB‐1) assessed Randomized Controlled Trials, while the Risk of Bias In Non‐randomized Studies of Interventions (ROBINS‐I) tool evaluated nonrandomized studies.

**Results:**

The review includes 11 articles assessing 579 patients. Excluding one study with 229 patients, of the remaining 550 patients, 338 (61.5%) were females. Treatments used were dopamine antagonists in 118 patients, antiepileptics in 198, corticosteroids in 134, IVIG in 7, and PE in 8 patients. Dopamine antagonists, particularly haloperidol, were the primary treatment choice, while valproic acid (VPA) was favored among antiepileptics. Prednisolone, a corticosteroid, showed promising results with weight gain as the only side‐effect. Our review emphasizes the importance of immunomodulators in SC, contrasting previous literature.

**Conclusion:**

Despite limitations, dopamine antagonists can serve as first‐line agents in SC management, followed by antiepileptics. The role of immunomodulators warrants further investigation for conclusive recommendations.

## Graphical abstract

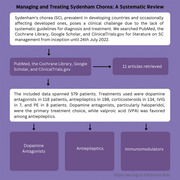

## 1 INTRODUCTION

Sydenham chorea (SC) is a neurological disorder that is a manifestation of acute rheumatic fever. The condition is associated with Group A Beta‐hemolytic *Streptococcus* (GAS) causing pharyngitis, taking approximately 6–8 weeks to develop (Beier & Pratt, 2023). It is most prevalent in early childhood, between the ages of 5 and 18, and manifests as purposeless and spontaneous movements (Beier & Pratt, 2023). Patients with SC can also present with mood disorders including ADHD, altered cognitive functioning, and schizophrenia (Punukollu et al., 2016).

A wide range of treatments for SC have been tested with variable efficacy. These include methods of immunosuppression, such as corticosteroids, among which prednisolone (PR) is the most prominently used. Other methods include administration of Intravenous Immunoglobulin (IVIG) and plasmapheresis (PE) (Ben‐Pazi et al., 2012), which has shown promising results in treating SC, particularly in cases where IVIG therapy has failed or there is a severe presentation of the disease (Miranda et al., 2015).

Owing to its pathogenesis, which is thought to involve dopamine receptor autoantibodies in the basal ganglia of the brain, there have been reports of usage of dopamine antagonists, such as haloperidol, pimozide, chlorpromazine, and sulpiride, with varying results (Ben‐Pazi et al., 2013). Haloperidol is among the most widely used neuroleptics in the treatment of SC due to its efficacy in reducing chorea. However, the drug is known for potentially serious side‐effects including extrapyramidal symptoms, tardive dyskinesia, and neuroleptic malignant syndrome in some patients (Rahman & Marwaha, 2023). Pimozide, on the other hand, has also been used in the treatment of SC with promising results, particularly in refractory cases (Harries‐Jones & Gibson, 1985). The use of pimozide, however, can cause extrapyramidal symptoms similar to those of haloperidol as well as ECG abnormalities, which can be severe, particularly in patients who are taking high doses for prolonged periods (Tueth & Cheong, 1993).

Antiepileptics, such as valproic acid, carbamazepine (CBZ), diazepam, phenobarbitone (PBB), and levetiracetam (LEV), have also been tested in clinical settings (Genel et al., 2002). Valproate has been shown to be an effective treatment for SC, with studies showing that it leads to a significant decrease in chorea symptoms (Dhanaraj et al., 1985). However, the drug can cause sedation, tremors, and weight gain, among other side effects. CBZ is another promising option among the treatments of SC, particularly in cases where other medications have failed (Genel et al., 2002).

Treatment of SC has three aspects: treating symptoms, preventing recurrence, and minimizing side‐effects. An effective treatment would have quick remission, low recurrence, and few to no side‐effects. As such, a balance of effectiveness and degree of adverse reactions is an important aspect to consider when weighing different treatments of SC. Current treatment of SC first involves administration of antibiotics for the GAS infection, which consists of intramuscular benzathine penicillin G. This also provides prophylaxis for SC while also reducing likelihood of recurrence (Gebremariam, 1999). The treatment of the chorea itself, however, is still being studied. It is, thus, essential to document the available data and conclude on the effectiveness of the different treatments for SC, so that patients can be treated with the best possible option for their condition. This study aims to bridge this gap by providing a comprehensive breakdown of the currently used methods of treating SC along with their expected side‐effects.

## 2 MATERIALS AND METHODS

**FIGURE 1:**
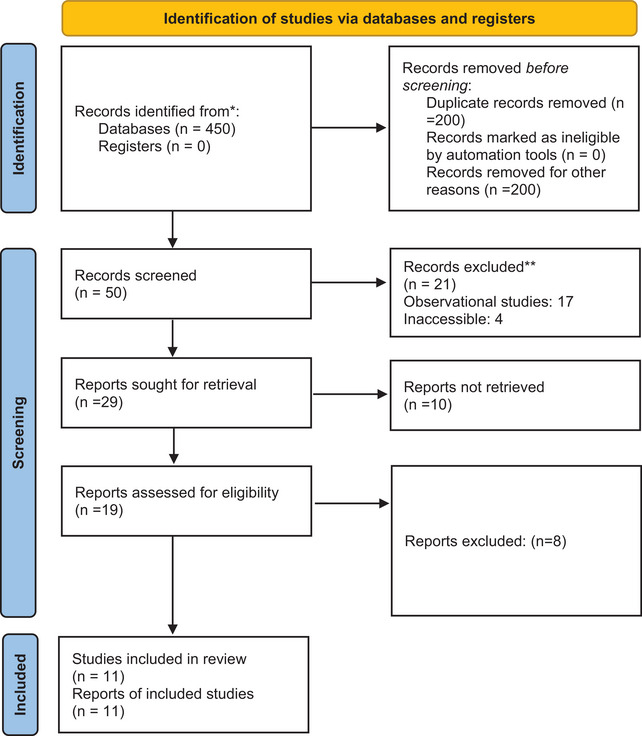
PRISMA updated guidelines for reporting systematic reviews.

This Systematic Review followed Preferred Reporting Items for Systematic Reviews and Meta‐Analysis (PRISMA) guidelines updated in 2020, illustrated in Figure 1 (Page et al., 2021).

### 2.1 Data sources and search strategy

Three authors (TGS, SHA, SW) independently conducted a thorough literature search on PubMed, the Cochrane Library, Google Scholar, and ClinicalTrials.gov from inception till 24th July 2022. The search was unrestricted by language and included keywords, “Sydenham's Chorea,” “treatment,” “antidepressants,” “antipsychotics,” “immunosuppressants,” “symptomatic treatment,” “plasmapheresis,” and “antibiotics.” Related terms, synonyms, and variant spellings were also incorporated with the Boolean operators “AND” and “OR.” The resulting literature was sorted and screened for duplicates following a more vigorous assessment by reading through titles, abstracts, and full texts. Bibliographies of recruited articles and similar reviews were screened as well for relevant data. After undergoing this process, any studies meeting our inclusion criteria were recruited for our study.

### 2.2 Inclusion and exclusion criteria

Following the literature search, all selected studies were screened via titles and abstracts for full‐text assessment. Four authors (TGS, STA, FN, SW) conducted a full‐length review of each article, and those which met our inclusion criteria were included in the final study. Any discrepancies were resolved at the discretion of a fifth reviewer (SHA). Our inclusion criteria were as follows: (1) Randomized controlled trials (RCTs) and cohort studies; (2) Studies exploring the treatment of SC; (3) Studies available in English. Our exclusion criteria were defined as follows: (1) Case studies and case series, letters, reviews, pilot studies, and protocols for clinical trials. (2) Studies not exploring the treatment of Sydenham's chorea. (3) Studies that were not available in English.

### 2.3 Data extraction and quality assessment 

The data extraction was performed by two independent investigators (FN, ST) with a third investigator (TGS). A spreadsheet was created, with the following data extracted: First author's name, study type, publication year, population characteristics, sample size, intervention(s) used, outcomes, and reported adverse effects. While some studies reported several outcomes, the primary outcomes included in our study were patient remission, disease recurrence, and drug response time as a measure of efficacious treatment.

All our included studies underwent a strict quality assessment. RCTs were assessed via the Cochrane Collaboration's Risk of Bias tool version 1 (Higgins et al., 2011), which consists of seven aspects including (1) random sequence generation, (2) allocation concealment, (3) selective reporting, (4) blinding of personnel, (5) blinding of outcome assessment, (6) incomplete outcome data, (7) any other potential sources of bias. Studies were thus assigned a low, high, or unclear risk of bias accordingly.

For nonrandomized studies in our article, the assessment was done via the Risk of Bias In Non‐randomized Studies ‐ of Interventions (ROBINS‐I) tool (2016). As such, all nonrandomized studies were scrutinized on seven domains: (1) confounding bias; (2) selection bias; (3) misclassification bias; (4) performance bias; (5) attrition bias; (6) detection bias; (7) outcome reporting bias. Each aspect was graded as low, high, or unclear risk of bias.

## 3 RESULTS

### 3.1 Literature search

Our electronic database search yielded 450 results. After removing duplicates and irrelevant studies, 50 underwent screening. These studies were then fully assessed via full‐text reviews, after which 11 fulfilled our inclusion criteria and thus remained (Araujo et al., 2002; Demiroren et al., 2007; Direk et al., 2020; Favaretto et al., 2020; Garvey et al., 2005; Gebremariam, 1999; Genel et al., 2002; Kulkarni & Anees, 1996; Orsini et al., 2022; Paz et al., 2006; Peña et al., 2002). The result of our literature search is summarized in the PRISMA flowchart, illustrated in Figure 1.

### 3.2 Study characteristics

Among our 11 included studies, two were RCTs (Garvey et al., 2005; Paz et al., 2006), while the remaining set consisted of nonrandomized studies (Araujo et al., 2002; Demiroren et al., 2007; Direk et al., 2020; Favaretto et al., 2020; Gebremariam, 1999; Genel et al., 2002; Kulkarni & Anees, 1996; Orsini et al., 2022; Peña et al., 2002).

Our included studies assessed a total of 579 patients. (Demiroren et al. (2007) reported the outcomes of 29 patients but did not include the male–female distribution in the final group. Among the rest of the 550 patients from the remaining ten studies, 338 (61.5%) were females. Patients in our studies received treatment from at least one of four major treatment groups. These were dopamine antagonists, antiepileptics, immunosuppressants (which were further divided into corticosteroids, IVIG, or PE), and control (placebo or no treatment). Apart from one study (Demiroren et al., 2007), our included studies clearly stated the distribution of treatment groups and assignment of patients. As such, the final treatment distribution of our included studies was dopamine antagonists in 118 patients, antiepileptics in 198, corticosteroids in 134, IVIG in 7, and PE in 8 patients. Table 1 shows the characteristics of our included studies.

**TABLE 1:** Study characteristics of included studies in this review

Study, Year	Study design	Patients characteristics	Drugs with dosage	Distribution of drugs	Outcomes	Side Effects
Direk et al. (2020)^*^	Retrospective	*N* = 140 *F* = 98 *M* = 42 Age (years)= 11.8 ± 2.8 (6.0–16.7) Hemichorea= 45 Chorea= 95 Duration of chorea until admission (days)= 28.2 ± 25.8	**Dopamine antagonist**=HLP **Antiepileptic**=VPA, LEV, and CBZ	HLP= 29 CBZ= 36 Na‐VPA= 60 LEV= 15	**Response Time**= HLP:1‐6M, CBZ: 2‐4W, VPA: 1‐2W **Response Time as 2^nd^ choice=** VPA: 1‐2W, CBZ 2‐4W, LEV 1‐6M **Remission Time=** Similar between all drugs	Mild Drowsiness= Na‐VPA: 2, CBZ: 4, HLP: 4 Increased Appetite= Na‐VPA:5 Nausea= CBZ: 3 Dizziness= CBZ: 4, HLP: 4
Araujo et al. (2002)^*^	Observational	*N* = 20 Age (years)= 8 (6.0–12.0) *F*= 13 M= 7	**Dopamine antagonist**= HLP **Antiepileptic**= VPA **Immunomodulators**= Prednisone **Others**= DZP and SLP	HLP= 12 VPA= 2 PR= 1 DZP= 5 SLP= 0	**Recurrence=**Symptomatic Treatment: 4, PR: 0	Sedation occurred in 1/3^rd^ people treated with symptomatic drugs
Demiroren et al. (2007)	Retrospective	*N*= 29	**Dopamine antagonist**= HLP and PMZ	HLP vs. PMZ	**Response Time**=HLP:14.5 ± 10.7d, PMZ: 29.5 ± 42.9d **Remission Time**=HLP 42.7 ± 29.9d, PMZ: 109.5 ± 115.5d **Total Time of drug use**=HLP: 51 ± 22.5d, PMZ: 84.3 ± 102.6d	HLP= 3 (dystonia, Parkinsonism, sleepiness, absentmindedness, and forgetfulness PMZ= 1 (sweating, sleepiness, headache, dry mouth, and numbness)
Favaretto et al. (2020)	Retrospective Observational	*N*= 30 Age^b^ (years)= 7.5 **Prednisone** Age^b^ (years)= 7.0 *F*= 13 *M*= 2 **Standard therapy (PMZ or VPA)** Age^b^ (years)= 9.0 *F*= 10 *M*= 5	**Immunomodulators**= PR (2 mg/kg/day) **Antiepileptic**= PMZ **Dopamine antagonist**= VPA	PR= 15 Standard care= 15	**Response Time**= PR: 4d, Standard therapy: 16d **Remission Time**= PR: 30d, Standard therapy: 125d **Relapse**= PR: 1, Standard therapy: 3	Not reported
Gebremariam (1999; 2006)	Comparison study	*N*= 18 Age (years)= 9.2 ± 2.63 (5–13.25) *M*= 10 *F*= 8		BPG vs. control	**Response**= No significant difference **Recurrence**= BPG 0, Control 10	
(1999) & Genel et al. (2002)	Prospective Comparison	*N* = 24 Age (years)= 11.3 ± 2.3 (5–14) Duration of complaints (days)= 24.6 ± 37.3 (2–180) *F* = 15 *M* = 9 **Sodium Valproate** Age (years)=12.4 ± 1.5 *F*= 5 *M*= 2 **Carbamazepine** Age (years)=10.9 ± 2.4 *F*=10 *M*=7	**Antiepileptic**= Na‐VPA (20 mg/kg/day), CBZ (15 mg/kg/day)	CBZ= 17 Na‐VPA= 7	**Response Time**= CBZ: 7.4 ± 8.2d, Na‐VPA: 8.0 ± 4.0d **Remission Time**= CBZ: 10.1 ± 8.5w, Na‐VPA: 6.7 ± 6.3w **Recurrence**= CBZ: 3, Na‐VPA: 1	None
( Paz et al. (2006)	RCT	*N*= 37 M:F= 1.3:1 Hemichorea= 14 Chorea= 23 **Prednisone** M:F=1.2:1 Age (years)= 9.3 ± 1.9 **Placebo** M:F= 1.5:1 Age (years)= 10.5 ± 2.1	**Dopamine antagonist**= HLP	PR=22 PLB=15 HLP=4 in PR, 7 in PLB	**Response Time**=PR: 1 week **Remission Time**=PR:54.3±23.81, PB: 119.9±84.21 **Recurrence**=PR: 4, PB: 3	Weight gain Cushingoid appearance
Kulkarni and Anees (1996)	Prospective study	*N* = 60 Age (years)= 11.1 (7–18) *F*= 36 *M*= 24	**Antiepileptic=** Na‐VPA (20 mg/kg/day), PBB (3 mg/kg/day) **Dopamine Antagonist=** HLP (0.05 mg/kg/day), CPZ (2 mg/kg/day) **Others=** DZP (0.2 mg/kg/day)	Na‐VPA= 8 PBB= 13 HLP= 17 CPZ= 9 DZP=3 PBB and CPZ	**Response**= PBB 13.7d, CPZ 17.9d, CPZ + PBB 21.8d, DZP 15d, HLP 12.6d, VPA 9.7d **Recurrence**= 13 (not specified)	None
Garvey et al. (2005) &	RCT	*N*= 18 *F*= 11 *M*= 7 Age= 10.2 ± 2.3	**Immunomodulators**= IVIG (1 g/kg), PE, PR	IVIG= 4 PE= 8 PR= 6	**Recurrence**= IVIG: 3, PE: 2	IVIG= nausea:2, vomiting: 2, headache: 2, Hepatitis C:1 PE=Vasovagal episode without syncope: 2, citrate‐induced circumoral paresthesias: 1, Gram‐negative sepsis with *Enterobacter cloacae*: 1 PR= weight gain
Orsini et al. 2022)	Retrospective	*N*= 171 *F*= 108 *M*= 63 Age= 9^b^	**Immunomodulators**= IVIG, CS **Antiepileptic**= VPA **Dopamine antagonist**= HLP	BZP= 151 CS= 59 IVIG= 3 CS + DA= 25 CS + AE= 22 AE= 18 DA= 8 AE + DA= 2	**Remission Time**= (unspecified) 6 months= 82 8 months= 80 **Recurrence**= 16 (unspecified)	N/A
Peña et al. (2002)	Comparison study	*N*= 32 *F*=10 *M*=8 Age= 11 ± 0.861 (11–15)	**Immunomodulators**= IVIG, CS **Antiepileptic**= VPA (20 mg/kg/day), CBZ (15 mg/kg/day) **Dopamine antagonist**= HLP (3 mg/day)	HLP= 6 VPA= 6 CBZ= 6	**Response Time**= HLP: 3 patients, 5 days **Remission Time (Complete recovery)**= VPA: 5 days, CBZ: 7 days **Recurrence**= CBZ: 3m, HLp: 10m	HLP= excessive somnolence: 1, dystonic reaction= 1, failed to improve: 1

F: Female, M: Male, N: Number, AE: Anti‐epileptics, DA: Dopamine Antagonists, HLP: Haloperidol, VPA: Valproic Acid, LEV: Levetiracetam, CBZ: Carbamazepine, PR: Prednisolone, DZP: Diazepam, SLP: Sulpiride, PMZ: Pimozide, BPG: Benzathine Penicillin G, PBB: Phenobarbital, IVIG: Intravenous Immunoglobulin, PE: Plasmapheresis, CS: Corticosteroids.

### 3.3 Risk of bias assessment

The results of the quality assessment of our included studies are summarized in Tables 2 and 3. Two of our studies (Garvey et al., 2005; Paz et al., 2006) were RCTs. While the study by Paz et al. (2006) was judged to be mostly low risk, the study by Garvey et al. (2005) returned mixed results. While the study showed adequate result reporting and outcome data, participants were not blinded to their respective treatment groups. Moreover, the study methodology was largely unclear, and the adequacy of random sequence generation could not be determined. Furthermore, in this study, patients in the IVIG group were given diphenhydramine chloride and acetaminophen to reduce adverse effects. This may have influenced the results.

**TABLE 2:** Risk of bias for included RCTS via Cochrane Collaboration's Risk of Bias tool version 2

Study	Random sequence generation (selection bias)	Allocation concealment (selection bias)	Blinding of participants and personnel (performance bias)	Blinding of outcome assessment (detection bias)	Incomplete outcome data (attrition bias)	Selective reporting (reporting bias)	Other Bias
Paz et al. (2006)	Low	Low	Low	Low	Low	Low	Low
Garvey et al. (2005)	Low	Unclear	Unclear	Unclear	Unclear	Low	Low

**TABLE 3:** The risk of bias assessment of included nonrandomized studies using ROBINS‐I tool

Study	confounding	Selection of participants	Classification of intervention	Deviation from intended intervention	Missing data	Measurement of outcome	Selection of reported results	Overall bias
Direk et al. (2020)	low	Low	low	low	low	Serious	low	low
Araújo APQC et al. (2002)	low	Moderate	Serious	low	low	low	low	low
Demiroren et al. (2007)	low	Serious	low	low	Serious	low	low	Moderate
Favaretto et al. (2020)	Low	Low	Low	Low	Moderate	Moderate	Low	Low
Gebremariam (1999)	Low	Low	Low	Low	Low	Low	Low	Low
Genel et al. (2002)	Low	Low	Low	Low	Low	Low	Low	Low
Kulkarni and Anees (1996)	Low	Severe	Low	Low	Low	Low	Low	Low
Orsini et al. (2022)	Low	Severe	Severe	Low	Severe	Low	Low	Severe
Peña et al. (2002)	Low	Low	Low	Low	Low	Low	Low	Low

### 3.4 Treatment regimens

#### 3.4.1 Dopamine antagonists

##### 3.4.1.1 Haloperidol

In a retrospective study from 2020, a total of 40 patients out of 140 chose haloperidol as their first drug of choice and decided to remain on it even after experiencing side‐effects (Direk et al., 2020). Haloperidol had the highest number of patients showing side‐effects, with four showing dizziness and four experiencing drowsiness (Direk et al., 2020). In another observational study, 12 patients out of 20 (60%) were prescribed haloperidol and 1 patient was given prednisone (5%), 2 were given valproate (10%), and 5 were given diazepam (25%), the shortest course of chorea occurred in prednisone (16 days). Sedation occurred in one‐third patients treated with symptomatic drugs (Araujo et al., 2002).

In an RCT, in 37 individuals, haloperidol was used in both the intervention (PR) and controlled group and reached remission in 54.3 ± 23.8 days in the intervention group in contrast to 119.9 ± 84.2 days in the placebo. However, it was difficult to pinpoint whether the efficacy of the treatment was due to PR or haloperidol (Paz et al., 2006). In another study, out of 60 patients, 17 patients were given 0.05 mg/kg/day of haloperidol, and they had a response time of 12.6 days, second only to the response time of those who were given valproic acid (9.7 days). There was a recurrence in 13 individuals, but not specified which drug‐associated group (Kulkarni & Anees, 1996). In a comparison study from 2002 between haloperidol, valproic acid, and CBZ, six patients were given 3 mg/day of haloperidol, of which three showed improvement in 5 days (the remaining three showed no signs of improvement), given haloperidol showed signs of excessive somnolence and dystonic reaction (Peña et al., 2002).

##### 3.4.1.2 Pimozide

In a retrospective observational study from 2020, pimozide acted as a standard therapy against PR, along with valproic acid, showing a response time of 16 days and a remission time of 125 days, both of which were distinctly longer than that of PR (4 and 30 days), furthermore, 3 out of 15 enrolled, to the standard group relapsed, compared to 1 in the PR group (Favaretto et al., 2020).

In a retrospective study comparing haloperidol and pimozide, the haloperidol group displayed a lesser duration of all the measured parameters with a response time of 14.5 ± 10.7 days, which was markedly less than the pimozide response time of 29.5 ± 42.9 days, haloperidol group reached remission earlier as well at 42.7 ± 29.9 days while it took 109.5 ± 115.5 days for pimozide group. However, notably, a greater presentation of side‐effects was seen in the haloperidol group (three patients) which included dystonia, parkinsonism, sleepiness, absentmindedness, and forgetfulness. While only one patient on pimozide showed side‐effects which included, sweating, sleepiness, headache, and dry mouth, all of which are mild and not a cause of concern (Demiroren et al., 2007).

##### 3.4.1.3 Chlorpromazine

This drug is not as frequently reported to provide symptomatic aid. In a prospective study from 1996, 9 out of 60 were given chlorpromazine, showing a remission time of 17.9 days while the patients who received phenobarbital along with chlorpromazine displayed a response rate of 21.8 days, the second longest response time of the drugs (Kulkarni & Anees, 1996).

##### 3.4.1.4 Sulpiride

Only one study reported Sulpiride as a treatment option for patients with SC. Although SLP was the second‐choice treatment of three patients, their specific outcomes were not reported. Sedation occurred in one‐third of patients and four had a recurrence. Four patients reported arthritis, three reported carditis, emotional lability was reported by five, and eight had severe disease; one was unable to walk while seven were admitted to the hospital (Araujo et al., 2002).

#### 3.4.2 Antiepileptics

##### 3.4.2.1 Valproic acid

In the study by Direk et al. (2020), 22 among the four drugs used, valproic acid (VPA) displayed the best results, with a response time of 1–2 weeks as a first choice as well as a second choice. Two patients on VPA reported mild drowsiness, five reported increased appetite. In the study by Araujo et al. (2002), two patients chose valproate as their first choice of treatment, while four selected it as their second‐choice drug. One additional patient chose VPA as their fourth‐choice drug, making the final tally of patients on VPA seven. The results of this study were not published with drug specificity; patients on symptomatic treatment were compared with PR therapy.

Kulkarni and Anees (1996) published a prospective study following the treatment outcomes of 60 patients on different therapies. Among these, eight patients had VPA (20 mg/kg/day) and had the best response time (at 9.7 days). Although 13 patients were reported to have had a recurrence of the disease, their groups were not specified. Orsini et al. (2022) retrospectively reported 171 patients, among which 18 were treated with VPA only, 2 with both VPA and HLP, and 22 with corticosteroids and VPA. The outcomes in this study were not specific; 82 patients had remission in 6 months and 80 in 8 months. Recurrence of the disease was reported in 16 patients (Kulkarni & Anees, 1996).

##### 3.4.2.2 Carbamazepine

Genel et al. (2002) detailed the course of 24 patients. Among these, 7 were treated with Na‐VPA (20 mg/kg/day) and 17 with CBZ. Patients on VPA had a response time of 8.0 ± 4.0 days whilst those on CBZ responded in 7.4 ± 8.2 days. Despite the similar response times, patients on VPA had better remission times at 6.7 ± 6.3 weeks compared to CBZ patients at 10.1 ± 8.5 weeks. One while three patients reported recurrences in VPA and CBZ therapy, respectively (Genel et al., 2002).

Another study highlighted the effect of CBZ in patients, comparing VPA and HLP. While all six patients on CBZ therapy had a complete recovery in 7 days, those on VPA had a remission time of 5 days. Moreover, patients on CBZ therapy had a recurrence after 3 months. While the response time was not reported for VPA, this drug had the best results among the reported outcomes, with an average remission time of 5 days for complete recovery and no recurrences and side‐effects. One patient on CBZ therapy had a recurrence after 3 months, while one on HLP had a recurrence 10 months after therapy. No patients on VPA had a recurrence (Peña et al., 2002).

##### 3.4.2.3 Diazepam

In the study by Araujo et al. (2002), diazepam was chosen by five patients as their first‐choice drug. The study was unclear regarding treatment outcomes, only reporting that four patients had a recurrence and one‐third of patients had sedation. Kulkarni and Anees (1996) reported a 15‐day response time from three patients on DZP (0.2 mg/kg/day). A total of 13 patients in this study had a recurrence, but their drug groups were not reported.

##### 3.4.2.4 Phenobarbitone (PBB)

In the study by Kulkarni and Anees (1996), PBB was used for treatment in two groups. One group received PBB (3 mg/kg/day) while the other received a combination of chlorpromazine (2 mg/kg/day) with PBB (3 mg/kg/day). Patients on PBB alone had an average response time of 13.7 days, which was less than that of the patients in the other group, who averaged a response time of 21.8 days. In this study, 13 of the patients had a recurrence. However, their groups, drugs, and details are not specified (Kulkarni & Anees, 1996).

##### 3.4.2.5 Levetiracetam

Only one study reported the use of LEV, A total of 15 patients were on LEV therapy. Initially, the study started with only two patients choosing LEV as their first‐choice drug. As the study progressed, however, an increasing number of patients choose to change their therapy of choice to LEV due to side‐effects from the other drugs. The two patients that chose LEV as their first choice continued their treatment course. While the study was not specific regarding the response time of LEV patients, the drug had a response time of 1 to 6 months as a second‐choice drug. The remission time between all drugs was similar (Direk et al., 2020).

#### 3.4.3 Immunomodulators

##### 3.4.3.1 Corticosteroids

Araujo et al. (2002) reported the outcomes of several drugs as a comparison between PR and symptomatic treatment. Though only one patient chose PR as their first‐choice drug, experiencing the shortest course of the disease at 16 days. Moreover, the patient reported no side‐effects and no recurrence of the disease while four patients on symptomatic treatment had a recurrence.

Favaretto et al. (2020) compared the efficacy of PR compared to standard care, PR (2 mg/kg/day) (n = 15) and standard therapy (n = 15), which entailed the use of VPA and PMZ. Patients on PR had a response time of 4 days, while standard care had 16. PR therapy patients also had a shorter remission time at 30 days compared to the 125 days of standard care. Only one patient in the PR group experienced a relapse while three were in the standard care group.

Paz et al. (2006) compared PR (2 mg/kg/day) against another drug group. In this study, 22 patients were given PR therapy. Four patients used HLP in conjunction with the experimental drug. The placebo group consisted of 15 patients, among which 7 used conjunctive HLP. Patients on PR displayed a quicker response to treatment, with the drug exhibiting a significant effect in week 1. The placebo group, in comparison, only displayed a significant effect after 2–3 weeks had passed. Moreover, the PR group had a significantly quicker remission time at 54.3 ± 23.81 days compared to the 119.9 ± 84.21 days in PLB patients. At week 12, patients in the PR group had achieved complete remission. The PLB group only had a Chorea Intensity Scale score improvement of −79.8. Although more patients in the PR group experienced relapse compared to PLB (4 and 3), this could be attributed to the greater sample size in the PR group and the higher number of patients on HLP therapy. Patients in the PR group experienced no severe adverse events, but weight gain and cushingoid appearance were reported.

Orsini et al. (2022) recorded 59 patients solely on corticosteroid therapy, 25 on combination therapy with corticosteroids and dopamine antagonists, and 22 on corticosteroids and antiepileptics. The study did not include group‐specific results, and 82 patients experienced remission in 6 months.

Garvey et al. (2005) conducted a RCT to compare the effects of PR, IVIG, and plasma exchange. A total of patients were assigned PR, eight to PE, and four to IVIG (1 g/kg). Patients on PR therapy displayed a slower response, with mean chorea severity scores of 9.4 ± 6.3 at 1‐month follow‐up, the lowest of the three groups. However, at a longer follow‐up time, PR therapy yielded the best results, with a mean chorea severity score of 0.7 ± 0.8, the lowest of all groups. Furthermore, PR therapy patients reported a maximum of 3 kg weight gain.

##### 3.4.3.2 IVIG and PE

The use of IVIG and plasma exchange (PE) was reported in only two studies. In Orsini et al. (2022), three patients were given IVIG. Their outcomes were not reported. A total of 16 patients from the study (which had 171 total patients) reported recurrence, and 82 patients had a remission time of 6 months. The RCT by Garvey et al. (2005) also included subgroups allocated IVIG and PE, with four and eight patients in their respective groups. The patients on IVIG therapy were given diphenhydramine chloride at a max dose of 300 mg/day and acetaminophen 10–15 mg/kg/dose to reduce adverse effects. IVIG displayed the best response at a 1‐month follow‐up with a mean chorea severity score of 3.8 ± 1.3. PE patients were not far behind, at 9.4 ± 6.3. In a long‐term follow‐up at 1 year, IVIG patients had a mean chorea severity score of 1.8 ± 1.3, while PE patients were at 2.1 ± 2.4. In the IVIG group, two patients experienced mild nausea, while reported vomiting and moderate‐severity headache. Two subjects in this group were Hepatitis C negative at study entry but were confirmed for anti‐hepatitis C antibodies at 3 months follow‐ups. In comparison, adverse effects in the PE group were mild. Patients experienced brief vasovagal episodes, and one experienced mild citrate‐induced circumoral paresthesia. Throughout the five or six procedures, patients' hematocrits dropped by an average of 13%. One patient, however, developed Gram‐negative sepsis with *Enterobacter cloacae* soon after her first exchange, which was determined to be related to procedure (Garvey et al., 2005).

## 4 DISCUSSION

With no specific diagnostic tests for a definitive confirmation, SC remains a clinical challenge in developing countries. In most cases, the illness subsides over time, however, in a few patients, the resolution may require medical interventions. However, despite its prevalence of at least 40% in patients with RF (Dhanaraj et al., 1985) and being one of the lead occasional outbreaks in developed countries as well, the literature is scattered, and no definite regimen has been formulated over the years. Hence, reiterating the need for comprehensive, systematically assessed data.

The underlying mechanism is the imbalance between the cholinergic and dopaminergic system, secondary to dysfunction of the corpus striatum, hence, the currently available option for the management includes dopamine antagonists, antiepileptics, and immunotherapy as some of the key medications.

Haloperidol, a dopamine antagonist, achieves its maximal effect when it blocks 72% of the dopamine receptors (Rahman & Marwaha, 2022). The literature has been reporting its efficacy for many years (Dornaus et al., 1984). This is in line with the included studies in this review. Direk et al. (2020) reported its effectiveness to combat SC, while Demiroren et al. (2007), recorded it to take the least number of days for recovery as well as for complete remission of SC. However, the study by Peña et al. (2002), mentioned that Na‐Valproate's action was quicker than HLP. Notably, complete remission was not achieved with either of the drugs, and the disease remitted after all, with a maximum timeframe of 10 months with HLP (Table 1). The drug has its side effects, including Parkinsonism, dystonia, weight gain, dyskinesia, and even oligomenorrhea, which have also been observed in the included studies (Rahman & Marwaha, 2022). Pimozide is one of the most widely accepted treatment modalities alongside Haloperidol. The drug is known to carry fewer side‐effects (Vasconcelos et al., 2019). This is consistent with one of the included studies in the review by Demiroren et al. (2007), which reported side‐effects in only one patient. However, its response time and efficacy have been a controversial discussion. The case report by Harries‐Jones and Gibson (1985) reported its treatment time to be only 2 days, while another case report by Shannon and Fenichel (1990), reported complete remission in 2 weeks This is contrary to the work of Demiroren et al. (2007) in which mean response, remission, and the total time were greater than that of HLP, as tabulated in Table 1.

Alongside dopamine antagonists, antiepileptic medications have been highlighted in the literature to manage SC's symptoms. In the case report by McLachlan (1981), Na Valproate showed promising results, with the response of the drug, recorded within a day and complete remission in a month. Similar results were replicated by Alvarez and Novak (1985), where dopamine antagonist failed, and shifting the patient to Na‐Valproate brought in the response within 24–48 h of drug initiation. This goes in consistence with the included articles in this review, where Na‐VPA showed an early response compared to CZP (Table 1), except for Genel et al. (2002), where no difference between the two was observed. LEV, another of the class, was only retrospectively observed in one article, where it showed promising results, hence, corroborating the previous findings and use of the drug in other chorea (Direk et al., 2020).

Since the disease is believed to be of autoimmune origin, some instances in literature proposed immunosuppression therapies including corticosteroids, IVIG, and PE. While the result in our review shows promising evidence, previous literature about their use has been controversial. This is because (i) all patients in SC group may not be of immune origin (Ben‐Pazi et al., 2013), and (ii) the adverse reactions to immune suppression therapies are well‐defined. Those outcomes can worsen in immune‐compromised demographics, thus, may be exacerbating risk of infections.

The review has some limitations. There was unavailability and scarcity of literature. Very limited data on the efficacy of different drugs, with a handful of trials and controlled work, were present alongside a small sample size. This points out the need of carrying out larger‐scale studies to determine a set regimen to treat SC. Moreover, our review includes observational studies to gather the maximum amount of evidence, which can lead to potential bias compared to a study with only RCTs.

## 5 CONCLUSIONS

The review highlights particular lacks in the treatment modalities, offered for the patients of Sydenham chorea. While the treatment choices varied from physician to physician and could be due to several reasons including side effects, cost availabilities, and severity, the subjectivity of the regimen should need a thorough audit, with higher‐level studies, that can, in the future, guide physicians to make appropriate subjective decisions.

## CONFLICT OF INTERESTS STATEMENT

The authors declare that there is no conflict of interest.

### PEER REVIEW

The peer review history for this article is available at https://publons.com/publon/10.1002/brb3.3035.
